# Divergence in social traits in Trinidadian guppies selectively bred for high and low leadership in a cooperative context

**DOI:** 10.1038/s41598-019-53748-4

**Published:** 2019-11-20

**Authors:** S. Dimitriadou, D. P. Croft, S. K. Darden

**Affiliations:** Centre for Research in Animal Behaviour, College of Life and Environmental Sciences, University of Exeter, Washington Singer Laboratories, Perry Road, Exeter, EX4 4QG UK

**Keywords:** Behavioural ecology, Animal behaviour

## Abstract

In many animal species, individuals with certain morphological, physiological, or behavioural traits may have a disproportionately large role in determining group behaviour. While most empirical studies of leadership have focused on behaviour of individuals exploring new environments or foraging, little is known about leading behaviour in other ecological contexts. Here, we use a selective breeding design in the Trinidadian guppy (*Poecilia reticulata*) to quantify the heritability of leadership in a cooperative context, and determine the behavioural traits associated with it. Firstly we found that phenotypic selection for high and low leadership (HL and LL, respectively) over three filial generations resulted in pronounced differences in leadership tendency with a moderate degree of heritability. In our assay of other social traits, LL males were more aggressive and sampled their social environment less than HL males, but HL and LL females did not differ in either aggressiveness or sociability. Traits such as boldness and exploratory tendency did not diverge between the two lines. Leading behaviour was thus associated with social traits in males, but not females; suggesting that there may be sex-specific mechanisms driving the emergence of leadership in this context. We discuss our findings in the context of the evolution of cooperation.

## Introduction

In mobile social groups, specific individuals may have a disproportionally large role in determining a group’s movements and the timing of these^1–3^. Leading a group during movement is thought to confer certain benefits, such as greater access to resources^4^, but can also be associated with higher risk of predation^1^, with individuals positioned at the front of a group experiencing a disproportional number of attacks^3,5,6^. An individual’s tendency toward leadership may thus be affected by the possession of particular traits (either morphological, physiological, or behavioural) that increase the propensity to act first in a given context see^2^. For example, leadership is thought to be positively associated with specific asocial behavioural traits, as has been shown with exploratory tendencies in zebra finches (*Taeniopygia guttata*)^7,8^ and barnacle geese (*Branta leucopsis*)^9^. Willingness to accept risk (i.e. boldness) has also been associated with leading: for example, in three-spined sticklebacks (*Gasterosteus aculeatus*), leading propensity in foraging tasks is negatively correlated with latency to leave a refuge^10–12^ and latency to recover from an aerial predation attack simulation^13^. These studies are part of a growing interest in leadership, its drivers and its effects on animal groups; however, most empirical studies focus on the leading behaviour of individuals moving in open fields e.g.^11,13^, exploring unfamiliar environments e.g.^14^, or on foraging trips^9,10,12^ and we still know very little about leading behaviour in other ecologically relevant and important contexts.

Here, we use the Trinidadian guppy (*Poecilia reticulata*) to quantify behavioural traits that are associated with leadership during cooperative interactions. To do this we test for a divergence in behavioural traits related to leading tendencies in guppies selectively bred for ‘high leadership’ and ‘low leadership’ phenotypes in a cooperative context. Guppies cooperate in the context of predator inspection, a behaviour in which an individual or a small group of individuals leaves the relative safety of the shoal to approach a potential threat in the vicinity, inspect it, and gather information on the level of threat posed, which is then transmitted to the rest of the shoal^15–18^. Individuals performing predator inspection are exposed to increased predation risk^19,20^, but can reduce this predation risk when inspecting a predator in cooperative partnerships^16,18,20,21^. Experimental work has shown that the lead individual during an inspection in these cooperative partnerships has a disproportionate risk of predation^20^. The Trinidadian guppy thus provides an excellent experimental system to investigate traits associated with leadership during cooperation.

In our study we also explore the heritability of the measured leadership behaviour. Heritability has been investigated for other behavioural traits across a range of species e.g.^22–25^, but the extent to which leadership tendencies in non-human animal groups are heritable remains unexplored. In humans a number of studies on twins have reported that self-reported leadership^26^, leadership role occupancy^27–29^, and emergent leadership^30^ are moderately to largely heritable, although the specific genes or processes involved in this heritability remain largely unknown (see^27^ for an exception). Leadership in non-human animals is not comparable to leadership measures used in humans, however, given these empirical studies, it is possible that other animal groups also show heritability for the propensity of certain individuals to disproportionally affect the behaviour of the group.

Given that leading tendencies have been associated with behavioural traits such as willingness to accept risk (i.e. boldness)^13,14^ and low sociability^31^ we might expect that higher leadership during predator inspection (more time in the lead position) would indeed be associated with these behavioural traits and in these directions. We predicted that by selectively breeding for the extremes of the leading behaviour continuum, in this case high and low leadership during predator inspection, other behavioural traits would also diverge. To test this, we selectively bred Trinidadian guppies for leading tendencies during predator inspection over three filial generations and determined the heritability of this trait. We then assessed sociability, aggressiveness, boldness and exploratory tendency in the third filial generation of these lines to determine the relationship between leadership and other behavioural traits.

## Results

### Selective breeding and heritability of leading behaviour

**Figure 1:**
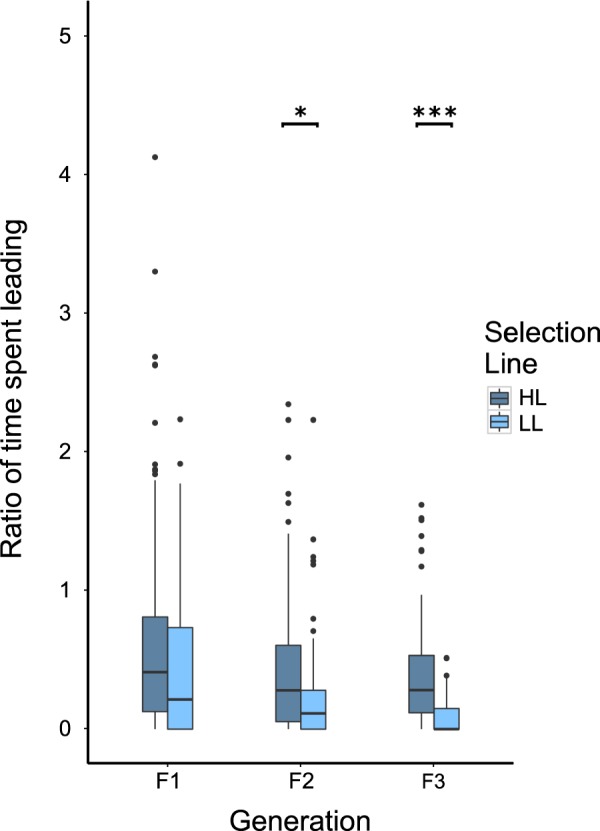
Median ratio of time spent leading across the three filial generations. Boxes represent the interquartile range (25th and 75th quartiles), and the horizontal lines represent the medians. The upper whisker extends to the largest value no further than 1.5 times the interquartile range (1.5*IQR), while the lower whisker extends to the smallest value within 1.5 times the interquartile range (Tukey boxplot). The dots represent outlying values. High Leaders (HL) and Low Leaders (LL) showed divergence in leading tendencies in F2, which was maintained and intensified in F3. *p < 0.05; ***p < 0.001.

Our two breeding lines (2 replicates per line with 15 breeding pairs per replicate) were set up using sexually mature, virgin female and male guppies (generation F0) selected from a population of 240 individuals tested for their leading propensity in a cooperative predator inspection paradigm (Supplementary Fig. S1). Experimental predator inspection paradigms have been extensively applied in this species, and usually involve the simulation of a cooperative partner with the use of mirrors e.g.^19,32,33^, which for some variants of the paradigm has been heavily criticised^34–37^. Although one variant of the mirror paradigm has recently been behaviourally validated with a comparison to performance with a live partner^38^ (physiological validation is still needed – see Oliveira *et al*.^39^), a constraint of such an assay, is that the ostensible partner will only ever match the investment of the focal and the focal can only ever match the investment of the partner. Here, we instead applied a novel method using a robotized guppy model as a standardized inspection partner where individuals could choose to overtake (i.e. assume the riskier inspection position) during inspections. We could then score the inspection behaviour (leading tendency) of focal individuals relative to this standard partner. We paired those with the highest leadership scores for the high leadership line (HL line) and those with the lowest leadership scores for the low leadership line (LL line). Subsequently, we tested the offspring of the breeding pairs over three filial generations and selectively mated the individuals with the highest and lowest scores within a line (Supplementary Fig. S2). We used linear mixed effects models (LME) to test for an effect of the selective process across the three generations as a function of sex (female and male), line (HL and LL) and generation (F1-F3) whilst controlling for replicate (1 and 2 within each line) and brood (full siblings). Here we found that the ratio of time spent leading (calculated as the time an individual spent assuming the riskier leading position relative to the time it would be expected to spend in that area of the tank if it were using space at random) across three filial generations (F1-F3) was affected by the interaction between the generation and the phenotypic selection line (‘Generation*Line’: F_2,125_ = 3.684, p = 0.028) (Fig. 1). Comparisons between HL and LL fish were carried out for each generation: these showed no difference in F1 (t_2,794_ = −1.366, p = 0.172). The two phenotypic selection lines began to diverge in generation F2, with HL fish spending more time ahead of the robotic model (assuming a higher risk) than LL fish (t_2,125_ = −3.163, p = 0.002); this difference was more pronounced in generation F3 (t_2,125_ = −6.423, p < 0.001) (Fig. 1). There was an overall trend for an interaction of sex and generation (‘Sex*Generation’: F_2,794_ = 2.761, p = 0.064) that did not reach statistical significance, and we did not detect any effect of sex or the interaction between line and sex on the time individuals spent leading. We calculated narrow sense heritability (h^2^) to estimate the variance in individual tendency to lead in this context due to additive genetic effects. Heritability of leadership tendency, estimated across sexes and phenotypic selection lines by fitting Markov Chain Monte Carlo generalised linear mixed models (MCMCglmm), was found to be 0.31 (0.21; 0.42). This is indicative of moderate heritability, consistent with heritability estimates of behavioural traits in general^40^.

### Sociability

**Figure 2:**
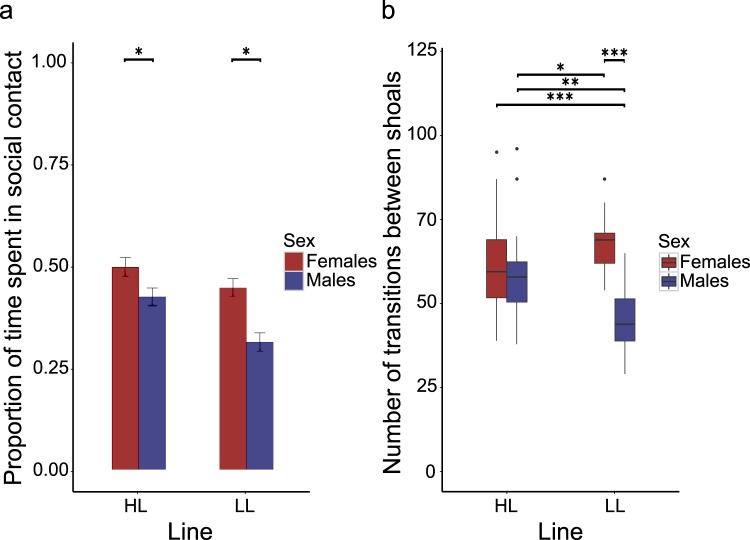
(**a**) Average proportion of time spent in social contact during the shoaling assay. Error bars represent standard error. Across phenotypic selection lines, females spent more time in social contact than males. (**b**) Number of transitions between stimulus shoals during the shoaling assay. Boxes represent the interquartile range (25th and 75th quartiles), and the horizontal lines represent the medians. The upper whisker extends to the largest value no further than 1.5 times the interquartile range (1.5*IQR), while the lower whisker extends to the smallest value within 1.5 times the interquartile range (Tukey boxplot). The dots represent outlying values. LL males performed less social sampling than any other experimental group. *p < 0.05; **p < 0.01; ***p < 0.001.

We used a shoaling assay with unfamiliar stimulus fish to measure the sociability (affiliative tendency) of predator naïve fish in the third filial generation (N = 96; n = 24 fish per replicate per line) reared to adulthood in mixed-sex groups within each selection line. Sociability in fish in laboratory conditions is usually measured by placing the focal fish in a novel tank with a group of conspecifics confined by a transparent partition and then measuring the time spent in close proximity to this stimulus shoal^41^, e.g.^42–44^. Here we used a modified version of standard sociability assays, introducing three, same-sex and size-matched stimulus shoals originating from an unselected population of fish, in spatially fixed clear cylinders which allowed for the transmission of visual and olfactory cues, and allowing single focal fish to move around freely in the experimental arena (Supplementary Fig. S3). With this design we were able to more closely simulate a naturalistic setting where individuals are able to join and leave small groups of fish, consistent with the fission-fusion character of this species^45^. We recorded their behaviour over a 25-minute period to extract three measures of sociability (i.e. attraction to conspecifics see^13,43,46^): (1) time spent within 2 body lengths of a shoal; (2) proportion of socializing spent in close (≤1 body length) versus far (≥2 body lengths) spatial proximity to shoals and (3) the amount of social sampling undertaken (number of shoal changes). Effects of phenotypic selection line (HL and LL), sex (female and male) and the interaction between line and sex on these measures while controlling for body length (standardized to account for size differences between the sexes) and replicate were tested by fitting linear mixed models to the data. We found that irrespective of phenotypic selection line, females spent a greater proportion of their time socializing compared to males (‘Sex’: F_1,86_ = 5.490, p = 0.021, Fig. 2a). There was a trend for HL fish to spend more time shoaling in close proximity to the stimulus shoals compared to LL fish that did not reach statistical significance (F_1,86_ = −1.733, p = 0.087). In addition, we found that there was an interaction effect of phenotypic selection line and sex (‘Line*Sex’: F_1,85_ = 14.357, p < 0.001) on the amount of social sampling that focals carried out. *Post hoc* analysis revealed that LL males performed less social sampling (fewer changes between stimulus shoals) than HL males, but that there was no difference in the comparison between LL and HL females (Fig. 2b).

### Aggressiveness towards conspecifics

**Figure 3:**
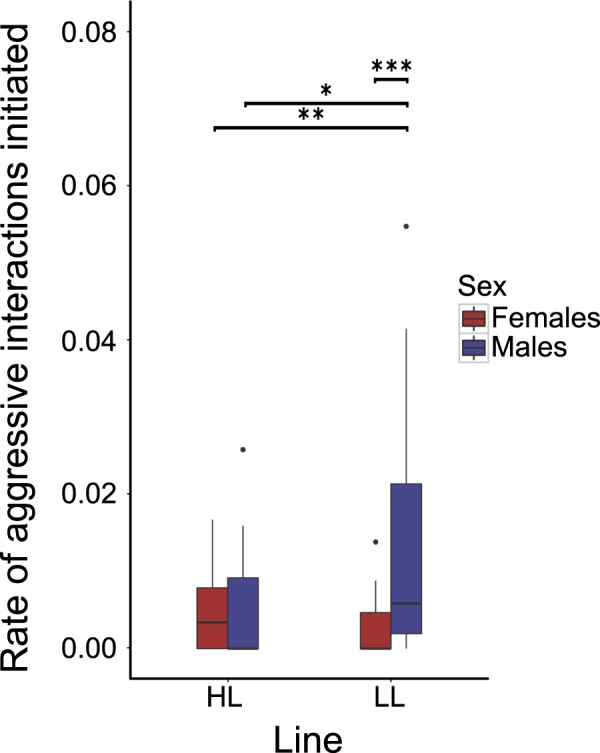
Rate of aggressive interactions initiated by females and males of the two phenotypic selection lines. Boxes represent the interquartile range (25th and 75th quartiles), and the horizontal lines represent the medians. The upper whisker extends to the largest value no further than 1.5 times the interquartile range (1.5*IQR), while the lower whisker extends to the smallest value within 1.5 times the interquartile range (Tukey boxplot). The dots represent outlying values. LL males exhibited higher aggression rates than any of the other experimental groups. *p < 0.05; **p < 0.01; ***p < 0.001.

We assayed fish in the third filial generation for their aggressiveness by quantifying the levels of aggression displayed by and towards single focal individuals from an unselected population of fish at a food patch in an open arena with two unfamiliar, size-matched, same-sex stimulus fish, using the ethogram by Seghers and Magurran^47^. Aggression assays similar to this, encouraging simultaneous feeding and allowing for an array of agonistic behaviours in an open arena, have been used in previous studies e.g.^47–49^. We fitted linear mixed effects models to the data to test for effects of phenotypic selection line (HL and LL), sex (female and male) and the interaction between line and sex on these measures, again while controlling for body length and replicate, and found that the rate of aggressive interactions initiated by the focal individual was affected by an interaction between its sex and the phenotypic selection line from which fish originated (‘Line*Sex’: F_1,84_ = 7.274, p = 0.009). Post hoc analysis revealed that LL males were more aggressive than HL males, but that there was no difference in the comparison between LL and HL females (Fig. 3). The rate of aggressive interactions directed toward the focal fish was not influenced by any of the factors included in the model (Supplementary Table S4).

### Boldness in the context of heightened risk of predation

**Figure 4:**
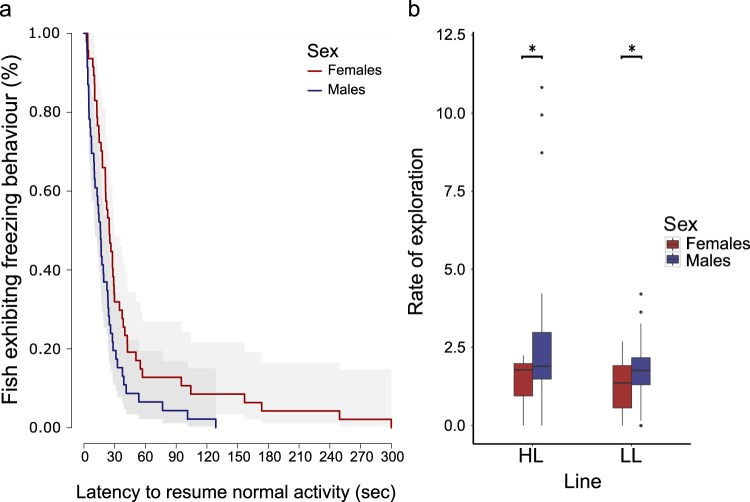
(**a**) Proportion of fish exhibiting freezing behaviour during a simulated aerial predation (grey areas represent 95% confidence intervals). Across phenotypic selection lines, males were faster to resume normal activity than females. (**b**) Rate of exploration [100*(zones explored)/time] exhibited by the sexes of each line. Boxes represent the interquartile range (25th and 75th quartiles), and the horizontal lines represent the medians. The upper whisker extends to the largest value no further than 1.5 times the interquartile range (1.5*IQR), while the lower whisker extends to the smallest value within 1.5 times the interquartile range (Tukey boxplot). The dots represent outlying values. Across phenotypic selection lines, males were faster explorers than females.

We assayed the third filial generation for boldness in the context of heightened risk of predation by simulating an aerial predation attempt and quantifying the latency to resume normal swimming behaviour following this attempt; an assay commonly used to determine phenotypic boldness e.g.^50,51^. We conducted a survival analysis using a Cox’s proportional hazards model to test for effects of phenotypic selection line of origin (HL and LL), sex (female and male) and their interaction on how quickly fish resumed normal swimming behaviour whilst again controlling for body length and replicate. We found that the latency to resume normal activity was affected only by sex (overall model: LRT_1,4.84_ = 17.97, p = 0.003), with males resuming normal activity faster (thus being bolder) than females [χ^2^_1_ = 6.15, p = 0.013, male odds of resuming normal activity were 2.2395 times that of females (95% CI: 1.1840 to 4.236), Fig. 4a, Supplementary Table S6].

### Exploratory tendency and levels of activity

We assayed the exploratory behaviour of our third filial generation in an experimental arena^52^ where we could also extract a proxy measure of activity, as our housing setup did not permit measuring general activity of focal individuals in their home tanks. We adapted an assay developed by Chapman and colleagues^52^, allowing fish to explore the arena individually for 12 minutes; the proportion of area explored per unit time (rate of exploration) and how much they moved around the arena (number of zone changes made) during this time was quantified (Supplementary Fig. S4). We tested for effects of phenotypic selection line of origin (HL and LL), sex (female and male) and their interaction on each variable whilst again controlling for body length and replicate using generalised linear mixed effects models. Sex was found to have a significant effect on the rate of exploration [(number of zones explored/time)*100], with males being faster explorers than females irrespective of phenotypic selection line of origin (F_1,77_ = 6.232, p = 0.015, Fig. 4b). However, HL and LL fish did not differ in their rate of exploration and we found no significant effect of the sex and line interaction. We also did not find an effect of phenotypic selection line or any of the other factors on our measure of activity (Supplementary Table S7).

### Social affiliation under heightened perception of predation risk

In our final paradigm we assayed whether a heightened perception of risk of predation differentially affected the tendency to affiliate with social partners in our third filial generation HL and LL fish. Fish where given the opportunity to shoal with either of two size-matched, conspecific, same-sex shoals, as well as to enter a neutral (non-shoaling) zone following visual exposure to a predator stimulus (static predator model) (Supplementary Fig. S5). Similar assays where social behaviour is observed after the presentation of a predator stimulus have previously been used in this study species e.g.^53^. We quantified their shoaling behaviour as the proportion of time they spent shoaling and tested for effects of phenotypic selection line of origin (HL and LL), sex (female and male) and their interaction whilst again controlling for body length and replicate, but also for the sociability of individuals (time spent socializing from the sociability assay). We did not detect any differences across phenotypic selection lines or as a function of any of our other model factors (Supplementary Table S8).

## Discussion

After just three generations of selective breeding for leading during a cooperative act, lines of Trinidadian guppies diverged in some, but not all assayed behavioural traits. Contrary to theoretical predictions, leading tendency was not linked to traits such as boldness or exploration, but appeared to be linked, in males, to behavioural traits expressed in a social context. Our results thus support a link between leading behaviour in a cooperative context and social, rather than asocial, traits, at least in males in this species.

Assuming a leading position in moving groups has been shown empirically to negatively correlate with social attraction and affiliative tendency^13,31,54^ and has also been theoretically demonstrated to surface as emergent behaviour in groups from individual traits such as ‘social indifference’^55^. We quantified three measures of sociability: shoaling tendency in close and far proximity to the stimulus shoals, and the extent to which individuals sampled their social environment. As expected, males were less sociable overall^56^, but HL and LL fish did not differ in their tendency to shoal (measured as proportion of time spent in social contact and in closest proximity to a shoal). Males of the two lines, however, differed in the amount of sampling of the social environment they carried out (number of transitions between stimulus shoals). Therefore, although males across lines had the same tendency to be in social proximity, LL males carried out less sampling of their social environment than HL males. This suggests that LL males may invest less in actively selecting preferred social partners than HL males. Social selectivity has not been extensively studied in non-human systems; there is, however, evidence from teleost fish that individuals can exhibit consistent individual differences in this trait. For instance, western mosquitofish (*Gambusia affinis*) have been found to exhibit individual differences in the extent to which individuals show a preference for any particular stimulus shoal; importantly, such individual differences have been found to be repeatable and positively correlated with individual sociability^43^.

When we quantified aggressiveness, we found that LL males initiated more aggressive acts near a food patch than HL males, while the rate of aggressive acts directed toward them remained the same. This pattern was not found in females, suggesting that males, but not females are divergent in this trait as a function of their propensity to lead during an inspection. Taken together, our results suggest that while there is no relationship between leading propensities and shoaling tendencies, this behavioural trait is negatively linked to social characteristics of what in the literature might be termed ‘faster’ behavioural types (i.e. less sampling of the environment and increased aggression)^57^. This is an exciting area for future research; in particular, it would be interesting to pursue questions regarding social mobility, which may indeed be linked to such ‘fast’ characteristics e.g.^58^.

Notably, neither boldness, exploration, nor social affiliation under predation threat were found to differ between HL and LL fish. This suggests that assuming the leading position during predator inspection is not strongly linked to differences in risk averse or exploratory behaviour. The literature to date suggests that individuals leading a moving group are subjected to disproportionally higher predation risk^1,5,6,59^; this is especially true for individuals performing predator inspection (as seen in three-spined sticklebacks, *Gasterosteus aculeatus*, where leading individuals can assume up to 90% of the risk^20^). Given our findings, it is possible that leadership in this context is not reflecting willingness to undertake risk across various contexts (a link that has been reported for leading tendency in moving shoals^13,14^), but rather reflects the different levels of investment individuals are ‘willing’ to make in this specific cooperative context (with more time spent leading amounting to a higher level of investment). When individuals either lead or lag during an inspection^17,33,60^; they manipulate the risk of predation they face^1,3,5,6,20^, alongside potential physiological costs associated with their respective level of threat^61–63^, and thereby their level of investment in the act relative to their partner. In such a case, the relationship between this behavioural trait and other specific social traits, such as sociability and aggressiveness, found here is perhaps indicative of a complex of traits contributing to a general social phenotype^64^ that may have important implications for the study of cooperative behaviour and its evolution amongst non-kin. An important next step for this work is to determine the net cost of leading versus lagging (i.e. cost – benefit) to more closely model these two behaviours during predator inspection in a game theoretic framework^65–67^.

Sex differences in the social behaviour of guppies have been well documented e.g.^50,68,69^. Our study corroborated these findings and emphasizes that the assayed social traits of females either are not related to leading behaviour, or perhaps more likely given some tendencies in our data, are less tightly coupled to this behavioural trait than in males and thus less prone to selection over such a small number of generations. Animals often show sex differences in the consistency of individual behavioural traits see^70^: for example, repeatability of a number of behaviours including leadership, and the context in which these are expressed, has been shown to differ between male and female zebra finches^8^. It is possible that such sex differences indicate differential levels of selection experienced by males and females^8,70^. In the Trinidadian guppy, where predator inspection behaviour of possible male mates affects female mate choice^71^, sexual selection on leading behaviour may have important evolutionary consequences.

Divergence in leading behaviour in a cooperative context following phenotypic selection appeared very rapidly in the selection process: fish in the HL and LL line showed no differences in leadership in generation F1; however, they showed differentiation in generation F2, which was maintained and intensified in generation F3. Anti-predator behaviour in the Trinidadian guppy has been shown to have at least some heritable component, with escape behaviour exhibiting genetic underpinnings and rapid evolution^72,73^. Our heritability estimates of leading tendencies ranged from 0.21 to 0.42, suggesting moderate heritability of this trait. To date, there are no studies explicitly looking at the heritable component of leadership in the Trinidadian guppy, but the field would greatly benefit from an examination of the mechanisms underlying this heritability.

## Conclusions

Following phenotypic selection for high and low leading behaviour in a cooperative context over three filial generations we demonstrated that male, but not female, guppies diverged in social traits that had not been explicitly selected for. Contrary to our predictions, leadership in predator inspection was not linked more generally to level risk adverse behaviour. Our work suggests that leading behaviour in predator inspection is, at least in males, closely linked to overall social behaviour and may have important implications for the study of cooperative behaviour in this species.

## Methods

### Ethics

All experimental protocols were approved by the U.K Home Office (Animal Scientific Licensing), and all experiments were undertaken under a U.K. Home Office project licence (30/3308) and personal licence (l002BDF3F). All methods were performed in accordance with the Animals (Scientific Procedures) Act 1986 (ASPA).

### Study subjects

#### Phenotypic selection lines

Generation F0: The fish used to set up two breeding lines were descendants of wild caught fish originating from a high predation site of the river Aripo on the island of Trinidad. The fish were housed at the University of Exeter, Department of Psychology (12 h light: 12 h dark cycle) in constant room temperature of 25 °C, and were fed with commercial flake and live food (*Artemia* sp) twice a day. At the first sign of sexual maturation (gonopodium formation), males were removed from the original housing tanks to male-only tanks, to ensure that females remained virgin.

Upon reaching sexual maturity, fish were tested once for their leading propensity in a cooperative context using a predator inspection assay in a custom-made arena. The assay was based on those previously used for this species e.g.^19,32,33^. The aim of such an assay is to measure the behaviour of focal individuals when given the opportunity to inspect a predator with an inspection partner. The arena consisted of two inspection lanes separated by clear Perspex. At the end of the focal inspection lane there was a predator compartment, separated by clear Perspex that allowed for the transmission of visual but not olfactory cues. A live predator was placed in the predator compartment. We used live pike cichlids [*Crenincichla alta*, a congenic species of *C. frenata*, a major predator of Trinidadian guppies in the wild – see Coleman & Kutty (2001) in Magurran^73^, and Weadick, Loew, Rodd, & Chang^74^]. A small plastic plant was placed at the other end of the inspection lane, to provide a refuge for the focal fish. The most widely used approach for simulating inspection partners is by placing a mirror (or mirrors) in the inspection lane so focal fish can inspect with their mirror image e.g.^16–18,32^. This approach has the caveat of a fish’s measured response being limited to distance and temporal measures relative to the predator, but not to the inspection partner. For instance, measures related to propensity to overtake an inspecting partner (which occurs in live-partner inspections) are not possible. Instead of using a mirror, we therefore simulated an inspection partner whose behaviour could be standardized using a realistic robotic model guppy, Robofish. This model was made of resin poured into silicone moulds of dead guppies. Colouration and other morphological features were achieved with the application of temporary tattoos onto the resin models (Inkwear, UK). Robofish was placed in one of the inspection lanes and followed a movement pattern that mimicked an inspecting fish, with the use of magnets moved by a stepper motor and pulley system as inspired by Faria and colleagues^75^.

The focal individual was placed in the inspection lane, with no visual access to the predator, and was given 10 minutes to acclimate. During this period, Robofish was behind the refuge of the other inspection lane and out of view of the focal fish. At the end of the acclimation period, the focal individual was gently herded with a hand net to the refuge area, and the opaque barrier obstructing visual access to the predator was lifted, signifying the start of the experimental trial. At the start of the trial, Robofish made an initial advance towards the predator (recruitment step) of 5 cm. Once the focal fish was recruited (left the refuge), Robofish continued the full movement pattern toward the predator over 19 seconds (10 cm distance intervals with 3 second stops); if the focal individual was not recruited within 1 minute of the completion of the recruitment step, Robofish continued the inspection movement pattern regardless. The trial ended after 5 minutes of exposure, at which time the focal individual was placed in an individual housing tank with visual access to 3 conspecifics.

The use of biomimetic robots in the study of animal behaviour has been increasing in the past decades, and can help us gain insight into leading and following behaviour e.g.^75–77^. It has recently been suggested that, in guppies, an individual’s tendency to interact with a biomimetic robot in an open field is comparable to interactions with live partners, thus reflecting individual ‘social responsiveness’ (i.e. tendency to respond to social cues)^78^. The ability to perceive and appropriately respond to cues from a social partner is an important element of predator inspection behaviour, especially with regards to overtaking and exchanging the lead position^18,60,79^.

All trials were video recorded and videos were then coded using Noldus Observer XT 10 (Wageningen, The Netherlands). We calculated leading tendency as the amount of time during the inspection trial that a focal fish spent in the lead position once Robofish was stopped (closest to the predator relative to Robofish, the position with the higher risk of predation see^20^) relative to the time the focal would be expected to spend in that part of the tank by chance alone if it were to use space directly proportional to its size (distance between Robofish and predator):

$${\rm{Expected}}\,{\rm{time}}\,{\rm{spent}}\,{\rm{leading}}=T\ast \frac{d}{D}$$

and

$${\rm{Leading}}\,{\rm{tendency}}=\frac{L}{Expected\,time\,spent\,leading}$$

where *T* is the time available for the focal to be in the lead position in seconds, *d* is the space available for the focal to be in the lead position (distance in centimetres between Robofish and the predator), *D* is the total space available for the focal fish to occupy (length in centimetres of the inspection lane), and *L* is the time in seconds the focal fish spent in the lead position. Inspecting individuals who showed the highest and lowest leading tendencies (30 males and 30 females per replicate of each phenotypic selection line) were selected and randomly placed into breeding pairs housed in 23 × 40 × 28.5 cm tanks in a flow-through circulation system.

Generations F1 and F2: The offspring of each breeding pair were housed in sibling groups in which a clear, perforated Perspex barrier was placed to separate males and females as they neared sexual maturity (see above). The barriers allowed visual and olfactory communication, but no physical contact. A month after sexual maturation, all the offspring from each breeding pair (generation F1) were tested using the same predator inspection assay described above. The 30 most and least cooperative inspecting males and females were selected from each replicate of a breeding line and randomly paired, generating the F2 generation (15 breeding pairs per phenotypic selection line per replicate). The same process was followed once more, resulting in the breeding pairs that produced the F3 generation. The first F3 generation broods underwent the same behavioural assay, with the most and least cooperative individuals being selected and randomly paired, to produce the 4^th^ filial generation, while the second broods were reared for testing (see below).

Test subjects (Generation F3): Generation F3 second broods were removed from their rearing tanks upon reaching sexual maturity, as described above. Fish were tagged with Visible Implant Elastomer (VIE; Northwest Marine Technology, USA), to allow for individual identification^45^, and placed in housing tanks containing individuals from different broods (but of the same selection line) in a sex ratio of 5 females: 3 males, to control for the effect of the social environment on behaviour. VIE has been shown not to affect social behaviour in this species^45^. Fish were housed in this manner for 95 days post tagging. At the end of this period, 12 males and 12 females from each selection line, originating from separate tanks, went through 5 behavioural assays exploring behavioural traits that may be associated with their cooperative phenotype. Each fish was tested once per day, with a 24-hour period between the assays. To avoid any carryover effects of previous tests on the behaviour during subsequent assays, the order of presentation of assays was randomised^80^.

Stimulus fish for behavioural assays: Some of the behavioural assays described below (sociability, aggressiveness and sociability under increased risk perception) required the use of stimulus fish. These consisted of Trinidadian guppies sampled from mixed-generation descendants of wild caught fish originating from the same sampling site on the Aripo river of Trinidad as the F0 generation fish. Stimulus fish were housed in four groups of 125 fish (100 females and 25 males), and were fed on the same diet (commercial flake, freeze-dried bloodworm and live food) as the groups of focal individuals, to avoid any confounding effects of different odour cues related to habitat exploitation^81^.

### Behavioural assays

#### Sociability

Three stimulus shoals each consisting of 3 same-sex fish unfamiliar to the focal were placed in clear cylinders (8 cm diameter), in a square tank. Surrounding each stimulus shoal were 2 shoaling zones [inner zone (12 cm diameter) and outer zone (14 cm diameter)]. The focal individual was introduced in a clear cylinder, and left for 5 minutes to acclimate. After the acclimation period, the cylinder was lifted and the focal fish was left free to swim and shoal with the stimulus shoals for 25 minutes. Each trial was video recorded and videos were analysed using Noldus Observer XT 10 (Wageningen, The Netherlands). Guppy social dynamics are characterised by high levels of fission-fusion over short time scales, with shoal encounters occurring on average every 14 s^45^. This paradigm allowed focal individuals to join and leave shoals and simulate shoal change opportunities as in the wild, but without the influence of the fission and fusion choices of others. The behavioural measures recorded were time spent in social isolation, time spent in each shoaling zone, and the number of transitions between different stimulus shoals.

#### Aggressiveness

To measure aggression towards unfamiliar individuals, two size-matched, same-sex conspecifics, unfamiliar with each other and the focal fish, were used. The stimulus and focal fish were introduced in a tank (12 × 19 cm), with a food patch placed in a clear cylinder. To habituate the fish to feeding from a food patch on the bottom of the tank, all fish were fed with commercial freeze-dried bloodworm from food patches placed on the bottom of their home tanks for 14 days prior to the start of the experimental period. After 5 minutes of acclimation, the clear cylinder was lifted and the fish were free to feed for 10 minutes. Trials were video recorded and aggressive interactions were coded using Noldus Observer XT 10 (Wageningen, The Netherlands). To identify aggressive interactions, we used the ethogram by Seghers and Magurran^56^. In summary, the aggressive behaviours scored were nipping, nudging, rapid approaching or chasing, circling or parallel swimming, tail beating and patch monopoly. The direction of these behaviours (whether they were initiated by or toward the focal fish) was also recorded.

#### Boldness

Individual boldness was measured using an aerial predation simulation paradigm^50,51^. Individuals were placed in a modified tank (12 × 19 cm), a part of which (12 × 8.5 cm) was sectioned by plastic mesh (drop area). Focal fish were given 10 minutes to acclimate. At the end of the acclimation period, when the fish were not expressing any escape behaviour such as erratic swimming or freezing, a weight was dropped in the drop area of the tank; the weight was tethered so that it broke the surface of the water but did not reach the bottom of the tank. Fish were then given a maximum of 5 minutes to resume normal swimming activity after the simulation of the aerial predation event. All trials were video recorded, and the latency to resume normal activity was recorded.

#### Exploratory tendency and activity

To measure exploratory tendency, we used an experimental setup similar to that of Chapman *et al*.^52^. An exploration tank with 5 corridors and a refuge area was used. The focal fish was placed in the refuge area for 3 minutes. At the end of this acclimation period, an opaque barrier that was obstructing access to the rest of the tank was lifted, and the focal individual was free to explore the area for 12 minutes. All trials were carried out under low light conditions, and the tank was backlit using an infrared LED array to facilitate tracking of movement. All trials were video recorded and videos were analysed using Noldus Observer XT 10 (Wageningen, The Netherlands). For the video analysis, the experimental arena was divided in 13 zones, not including the refuge area, and the total number of zones visited was recorded for each fish. We also calculated an exploration rate as the number of zones visited/duration of the test (seconds) after entering zone 2*100, which meant that individuals only received an exploration score greater than zero if they ventured into a part of the arena that could not be viewed from the refuge area.

#### Sociability under heightened perception of predation risk

Shoaling is thought to be a mechanism of decreasing risk of predation: larger shoals of inspecting fish are thought to provide more safety because of their increased ability to detect predators^82^, increased predator avoidance^83^, and the dilution of risk^84^. Guppy populations under high predation risk show higher shoaling tendency and form more cohesive shoals than those under relaxed predation regimes^85,86^. As shoaling acts to reduce risk of predation, and the evolutionary response to intensive predation is increased shoaling tendency, we would expect more risk-sensitive individuals to increase their shoaling tendency when the perceived risk of predation is high. Sociability following predator exposure was measured using a modified tank with 2 stimulus shoal compartments, and a choice compartment (Supplementary Fig. S5). Three size-matched, same-sex conspecifics, not previously encountered by the focal fish were placed in each stimulus shoal compartment, and were left for 5 minutes to acclimatise. The focal individual was then placed in the choice compartment, and was left for 10 minutes to acclimatise. At the end of this period, an opaque barrier obstructing visual access to a spatially fixed predator model of *Crenicichla frenata*, a guppy predator commonly found in high predation habitats (total length: 12 cm), was lifted, and the stimulus fish was exposed to this model predator for 1 minute. At the end of the exposure, the barrier was lowered again, once more obstructing visual access to the predator model, and the behaviour of the focal fish was video recorded for 10 minutes. All videos were analysed using Noldus Observer XT 10 (Wageningen, The Netherlands). The behavioural measures recorded were time spent in close proximity (within 2 body lengths) to each of the stimulus shoals.

### Statistical analysis

All statistical analyses were carried out in R v 3.2^87^. To test the effect of the phenotypic selection process on the fish of the filial generations (generations F1–F3) we analysed the ratio of time spent leading by fitting linear mixed effects models (LME) in the ‘nlme’ v 3.1–127 package^88^. The model included Line (High/Low Leaders) + Sex (Male/Female) + Generation (F1–F3) + Line*Sex + Line*Generation + Sex*Generation. Replicate and Brood were introduced as nested random effects (Replicate/Brood) with random intercepts. Our initial analysis showed that the response variable was heteroskedastic, with different variance structures for the different levels of each fixed factor; to overcome this, we used different variance structures for each level of each fixed factor^89^. Fish that did not leave the refuge area, and therefore did not perform a predator inspection, were excluded from the analysis.

The effect of the leadership phenotype on the behavioural measures assessed by the tests performed on subsequent broods of F3 fish was analysed by fitting linear mixed effects models in the ‘nlme’ v 3.1–127 package^88^. In many instances we observed heteroscedasticity of the response variable, which was resolved by adjusting the variance structure of the model^89^. Latency to resume normal swimming during simulated aerial predation was analysed using survival analysis^90^ using the ‘survival’ v 2.37–7R package^91^. We used a Cox’s proportional hazards model on the response variable (censored = 1). The ‘frailty’ function (gamma distribution) was used to add ‘Replicate’ as a random effect in the model. To examine the effect of the leadership phenotype on the percentage of area explored in the exploratory tendency assay (exploration index), we used generalised linear mixed effects models (family = beta, link = logit) in the ‘glmmADMB’ package v 12^92^. In all cases the model included Line (High/Low Leaders) + Sex (Male/Female) + standard body length (calculated as a z-score separately for males and females) + Line*Sex. When analysing sociability under heightened perception of risk of predation, we included the social tendency measured in the sociability assay (time spent socializing), in order to control for any differences in overall sociability (irrespective of predator exposure). Models had random intercepts and included Replicate as a random effect. Heritability was estimated by fitting Markov Chain Monte Carlo generalised linear mixed effects models (MCMCglmm) in the ‘MCMCglmm’ package v 2.22.1^93^.

## Supplementary information

Supplementary material

## Data Availability

All data generated and analysed in this study are included in this article and its supplementary files.
