# Supplementary material for: Divergence in social traits in Trinidadian guppies selectively bred for high and low leadership in a cooperative context
Source: Sci Rep. 2019 Nov 20;9:17194. doi: 10.1038/s41598-019-53748-4 (PMC6868185; doi:10.1038/s41598-019-53748-4)
Supplement: Supplementary file 1 — Supplementary material [file 41598_2019_53748_MOESM1_ESM.pdf]

Divergence in social traits in Trinidadian guppies selectively bred for high and low leadership in a cooperative context

Dimitriadou, S., Croft, D. P., and Darden, S. K.

SUPPLEMENTARY FIGURES

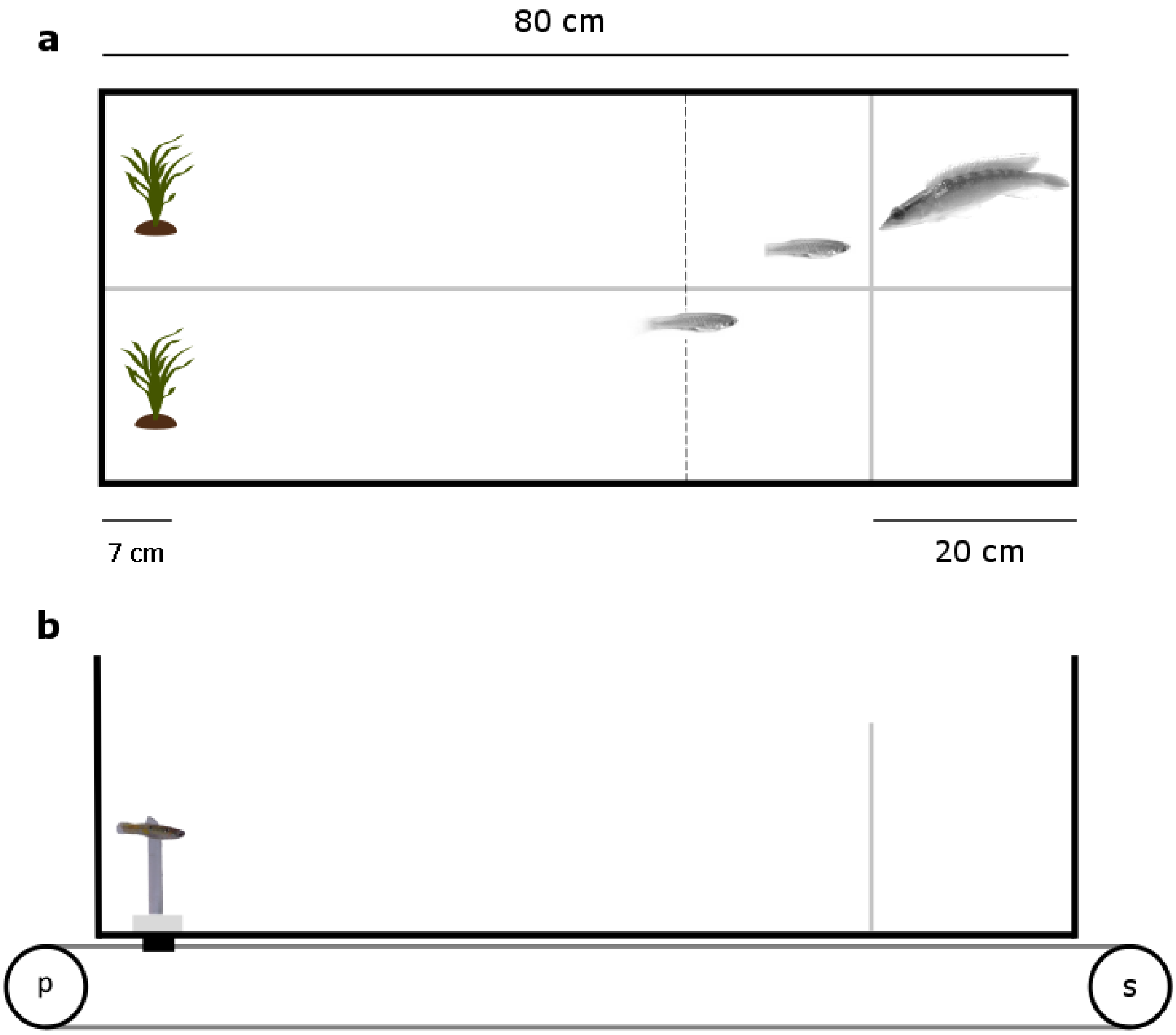

Figure S1. Top (a) and side (b) view of the experimental setup for the predator inspection assay. a: we used a standard predator inspection tank (80x31x60 cm) with two inspection lanes divided by clear Perspex. Live predators were placed in a predator compartment (right) that

allowed for the transmission of visual cues. Cooperation was simulated with Robofish (bottom lane) that always assumed the same distance from the predator (broken line). The time the focal fish (top lane) spent ahead of Robofish was used to calculate the ratio of time spent leading. b: Robofish was mounted on a glass rod and was moved with the use of two magnets, one inside and one underneath the tank. The magnet underneath the tank was attached to a timing belt and was moved with the use of a pulley (p) and a stepper motor (s).

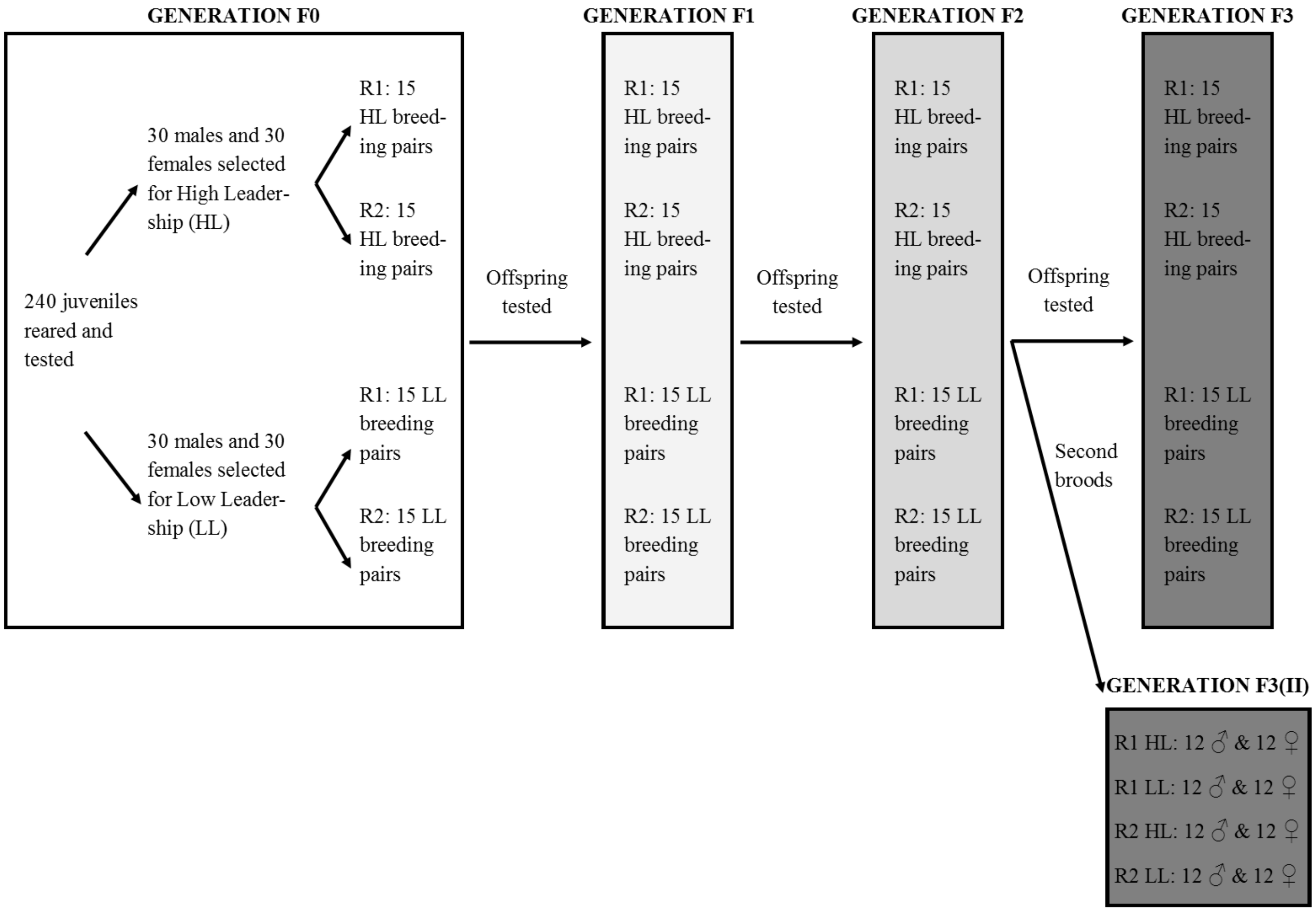
GENERATION F0
240 juveniles reared and tested
30 males and 30 females selected for High Leader-ship (HL)
R1: 15 HL breed-ing pairs
R2: 15 HL breed-ing pairs
30 males and 30 females selected for Low Leader-ship (LL)
R1: 15 LL breeding pairs
R2: 15 LL breeding pairs
Offspring tested
GENERATION F1
R1: 15 HL breed-ing pairs
R2: 15 HL breed-ing pairs
R1: 15 LL breeding pairs
R2: 15 LL breeding pairs
Offspring tested
GENERATION F2
R1: 15 HL breed-ing pairs
R2: 15 HL breed-ing pairs
R1: 15 LL breeding pairs
R2: 15 LL breeding pairs
Offspring tested
Second broods
GENERATION F3
R1: 15 HL breed-ing pairs
R2: 15 HL breed-ing pairs
R1: 15 LL breeding pairs
R2: 15 LL breeding pairs
GENERATION F3(II)
R1 HL: 12 ♂ & 12 ♀
R1 LL: 12 ♂ & 12 ♀
R2 HL: 12 ♂ & 12 ♀
R2 LL: 12 ♂ & 12 ♀

Figure S2. Overview of the breeding design for the phenotypic selection lines. Each filial generation (F1-3) comprised 60 breeding pairs (30 HL and 30 LL). Forty-eight males and females from second broods of the F3 generation [generation F3(II)] underwent behavioural testing for boldness during simulated aerial predation, exploratory tendency, aggressiveness, sociability and sociability following predator exposure.

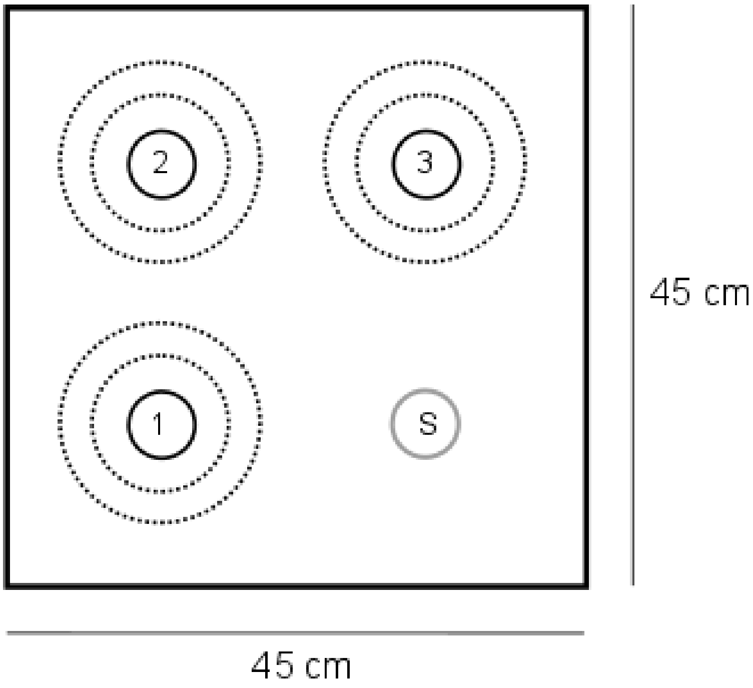

Figure S3. Top view of the experimental setup for measuring shoaling tendency. The numbered circles mark the position of the stimulus shoals (placed in clear cylinders). The dotted circles denote the two shoaling areas (inner zone (12 cm diameter) and outer zone (14 cm diameter)). S marks the point of introduction of the focal individual in the experimental arena.

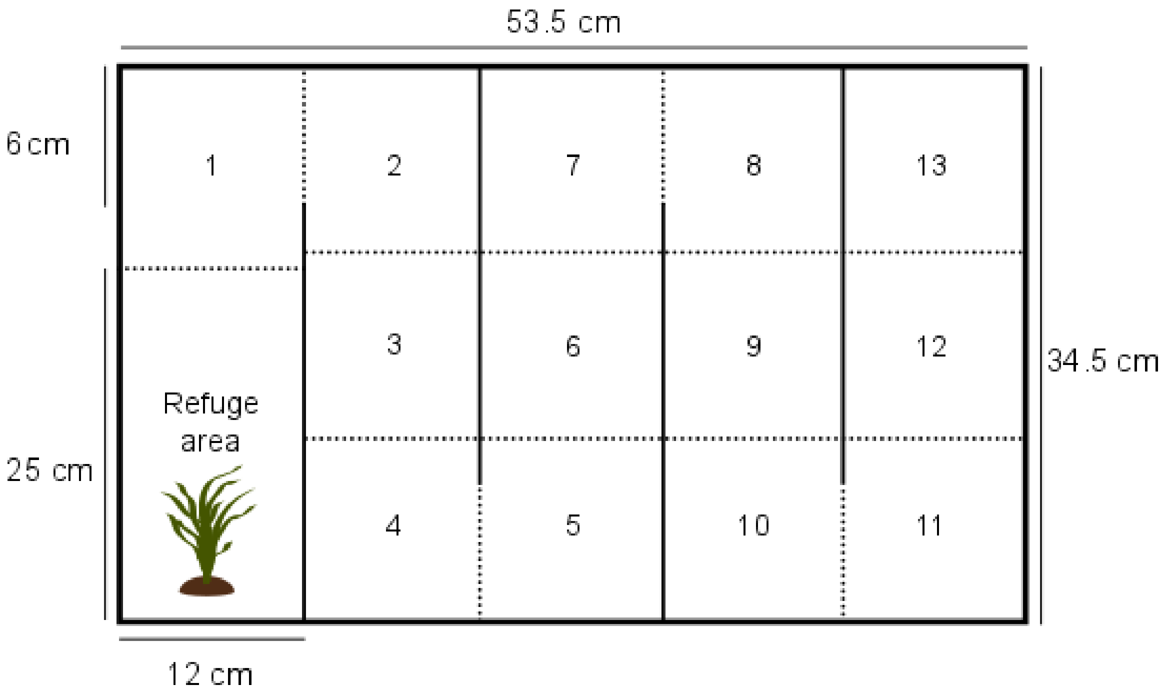

Figure S4. Experimental setup for the exploration assay (top view). The tank consisted of 5 parallel corridors, divided in 13 zones and the refuge area. The black lines depict the opaque maze barriers, while the dotted lines denote the zones. The number of zones visited was used to calculate the rate of exploration for each fish.

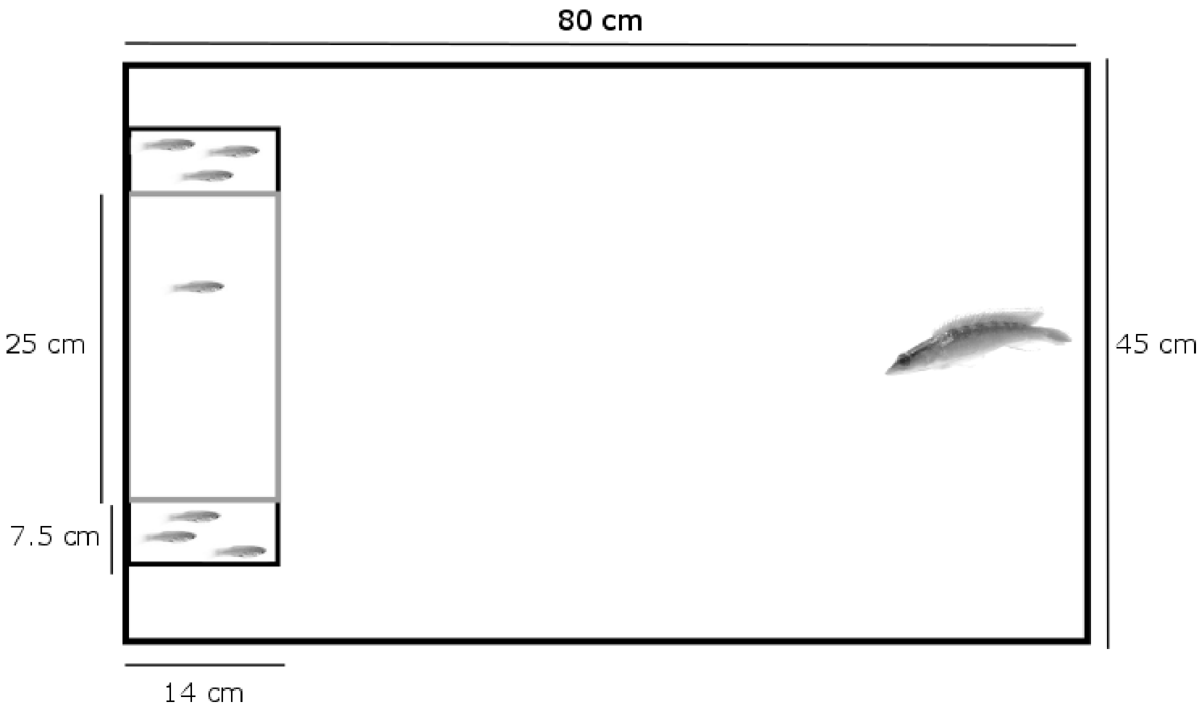

Figure S5. Top view of the experimental setup for the assay of shoaling tendencies after predator exposure. The focal fish is at the left, with one stimulus shoal on either side. On the right is the predator model.

SUPPLEMENTARY TABLES

Table S1. Marginal effects of Line, Sex and Generation on the ratio of time spent leading. LME with no model selection. Statistically significant factors are shown in bold.

		Estimate	Standard error	df	t value	p value
**Intercept**		**0.526**	**0.065**	**794**	**8.080**	**<0.001**
Line	HL	0	-	794	-	-
	LL	-0.072	0.069	794	-1.037	0.300
Sex	Females	0	-	794	-	-
	Males	0.038	0.063	794	0.608	0.543
**Generation**	**F1**	**0**	**-**	**125**	**-**	**-**
	**F2**	**-0.200**	**0.072**	**125**	**-2.777**	**0.006**
	**F3**	**-0.157**	**0.068**	**125**	**-2.305**	**0.023**
**Line*Generation**	**HL – F1**	**0**	**-**	**125**	**-**	**-**
	**LL – F2**	**-0.080**	**0.085**	**125**	**-0.926**	**0.356**
	**LL – F3**	**-0.206**	**0.081**	**125**	**-2.551**	**0.012**
Sex*Generation	Females – F1	0	-	794	-	-
	Males – F2	0.073	0.068	794	1.087	0.278
	Males – F3	-0.039	0.062	794	-0.620	0.535
Line*Sex	HL – Female	0	-	794	-	-
	LL – Male	-0.039	0.046	794	-0.838	0.402

Table S2. Marginal effects of Line, Sex and standard body length on the proportion of time spent shoaling, and the proportion of time shoaling spent in the inner shoaling area. LME with no model selection. Statistically significant factors are shown in bold.

**Measure**			**Estimate**	**Standard error**	**df**	**t value**	**p value**
Time spent in social isolation (%)	**Intercept**		**0.504**	**0.023**	**86**	**21.767**	**<0.001**
	Line	HL	0	-	86	-	-
		LL	0.051	0.032	86	1.600	0.113
	**Sex**	**Females**	**0**	**-**	**86**	**-**	**-**
		**Males**	**0.074**	**0.032**	**86**	**2.343**	**0.021**
	Standard length (z-score)		-0.003	0.012	86	-0.276	0.784
	Line*Sex	HL – Female	0	-	86	-	-
		LL – Male	0.058	0.045	86	1.289	0.201
Shoaling time spent in inner shoaling area (%)	**Intercept**		**0.531**	**0.032**	**86**	**16.796**	**<0.001**
	Line	HL	0	-	86	-	-
		LL	-0.048	0.028	86	-1.733	0.087
	Sex	Females	0	-	86	-	-
		Males	-0.014	0.027	86	0.504	0.616
	Standard length (z-score)		-0.003	0.010	86	0.286	0.776
	Line*Sex	HL – Female	0	-	86	-	-
		LL – Male	-0.039	0.039	86	1.002	0.319

Table S3. Marginal effects of Line, Sex and standard body length on the number of transitions between stimulus shoals. LME with no model selection. Statistically significant factors are shown in bold.

		Estimate	Standard error	df	t value	p value
**Intercept**		**61.134**	**2.438**	**85**	**25.100**	**<0.001**
**Line**	**HL**	**0**	**-**	**85**	**-**	**-**
	**LL**	**7.422**	**3.570**	**85**	**2.079**	**0.041**
Sex	Females	0	-	85	-	-
	Males	-3.609	3.494	85	-1.033	0.305
**Line*Sex**	**HL – Female**	**0**	**-**	**85**	**-**	**-**
	**LL – Male**	**-19.049**	**5.027**	**85**	**-3.789**	**<0.001**

Table S4. Marginal effects of Line, Sex and standard body length on the rate of aggressive interactions initiated and received by the focal fish. LME with no model selection. Statistically significant factors are shown in bold.

		Estimate	Standard error	df	t value	p value
**Intercept**		**$4.924*10^{-3}$**	**$9.677*10^{-3}$**	**84**	**5.08554**	**<0.001**
Line	HL	0	-	84	-	-
	LL	$-2.208*10^{-3}$	$1.383*10^{-3}$	84	-1.59664	0.114
Sex	Females	0	-	84	-	-
	Males	$9.893*10^{-5}$	$2.945*10^{-3}$	84	0.03354	0.973
Standard length (z-score)		$1.767*10^{-6}$	$6.626*10^{-4}$	84	0.00267	0.998
**Line*Sex**	**HL – Female**	**0**	**-**	**84**	**-**	**-**
	**LL – Male**	**$1.110*10^{-2}$**	**$4.064*10^{-3}$**	**84**	**2.69701**	**0.009**
**Intercept**		**$6.578*10^{-3}$**	**$1.825*10^{-3}$**	**84**	**3.60359**	**<0.001**
Line	HL	0	-	84	-	-
	LL	$1.205*10^{-3}$	$3.927*10^{-3}$	84	0.30688	0.815
Sex	Females	0	-	84	-	-
	Males			84		
Standard length (z-score)		$1.037*10^{-3}$	$1.057*10^{-3}$	84	0.98093	0.329
Line*Sex	HL – Female	0	-	84	-	-
	LL – Male	$3.780*10^{-3}$	$5.329*10^{-3}$	84	0.70928	0.480

Table S5. Post hoc analysis for the ‘Sex*Line’ interaction on the rate of aggressive interactions initiated. Pairwise least squares means comparisons. Statistically significant contrasts are shown in bold.

Contrast	Estimate	Standard error	z value	p value
Males HL – Females HL	$9.922*10^{-5}$	$2.381*10^{-3}$	0.042	0.999
Females LL – Females HL	$-3.691*10^{-5}$	$1.438*10^{-3}$	-1.536	0.397
**Males LL – Females HL**	**$8.852*10^{-3}$**	**$2.789*10^{-3}$**	**3.174**	**0.007**
Females LL – Males HL	$-4.683*10^{-4}$	$2.492*10^{-3}$	-0.926	0.779
**Males LL – Males HL**	**$8.753*10^{-3}$**	**$3.453*10^{-3}$**	**2.535**	**0.049**
**Males LL – Females LL**	**$9.221*10^{-3}$**	**$2.885*10^{-3}$**	**3.834**	**<0.001**

Table S6. Hazard ratios for Line, Sex and standard body length on the latency to resume normal activity after an aerial predation attack simulation. Cox’s proportional hazards model. Statistically significant factors are shown in bold.

		Exp(coeff.)	Exp(-coeff)	Lower 95%	Upper 95%	p value
Line	HL	0	-	-	-	-
	LL	1.1226	0.8908	0.5990	2.104	0.720
**Sex**	**Females**	**0**	**-**	**-**	**-**	**-**
	**Males**	**2.2395**	**0.4465**	**1.1840**	**4.236**	**0.013**
Standard length (z-score)		1.1916	0.8392	0.9615	1.477	0.110
Line*Sex	HL – Female	0	-	-	-	-
	LL – Male	0.7101	1.4083	0.2947	1.711	0.450

Table S7. Marginal effects of Line, Sex and standard body length on the rate of exploration and the rate of zone transitions during exploration. GLMM with no model selection. Statistically significant factors are shown in bold.

Measure			Estimate	Standard error	df	t value	p value
Rate of exploration	**Intercept**		**1.424**	**0.269**	**77**	**5.29445**	**<0.001**
	Line	HL	0	-	77	-	-
		LL	-0.103	0.307	77	-0.335	0.739
	**Sex**	**Females**	**0**	**-**	**77**	**-**	**-**
		**Males**	**1.462**	**0.586**	**77**	**2.496**	**0.015**
	Standard length (z-score)		0.085	0.125	77	0.681	0.498
	Line*Sex	HL – Female	0	-	77	-	-
		LL – Male	-1.011	0.673	77	-1.502	0.137
Rate of zone transitions	**Intercept**		**0.113**	**0.024**	**78**	**4.596**	**<0.001**
	Line	HL	0	-	78	-	-
		LL	$-2.895*10^{-4}$	0.035	78	-0.008	0.993
	Sex	Females	0	-	78	-	-
		Males	0.057	0.034	78	1.653	0.102
	Standard length (z-score)		-0.010	0.012	78	-0.814	0.418
	Line*Sex	HL – Female	0	-	78	-	-
		LL – Male	-0.022	0.048	78	-0.463	0.644

Table S8. Marginal effects of Line, Sex and standard body length on the proportion of time spent shoaling after predator exposure. LME with no model selection. Statistically significant factors are shown in bold.

		Estimate	Standard error	df	t value	p value
**Intercept**		**0.593**	**0.097**	**80**	**6.109**	**<0.001**
Line	HL	0	-	80	-	-
	LL	-0.015	0.061	80	-0.240	0.811
Sex	Females	0	-	80	-	-
	Males	-0.050	0.063	80	-0.793	0.431
Standard length (z-scores)		-0.185	0.023	80	-0.794	0.430
Social tendency		0.133	0.184	80	0.722	0.472
Line*Sex	HL – Female	0	-	80	-	-
	LL – Male	0.101	0.091	80	1.106	0.272
